# A toehold-triggered switchable three-way junction protective nanoprobe for RNase H-assisted HBV rcDNA detection

**DOI:** 10.1186/s12951-026-04344-y

**Published:** 2026-04-10

**Authors:** Jingyi Si, Suo Lv, Yifan Gao, Zhenzhou Yang, Wen Liang, Xizhong Shen, Gang Liu, Shao Su, Changfeng Zhu

**Affiliations:** 1 Department of Gastroenterology and Hepatology, Zhongshan Hospital, Fudan University, Shanghai, China; 2 State Key Laboratory of Organic Electronics and Information Displays & Jiangsu Key Laboratory for Biosensors, Institute of Advanced Materials (IAM), Nanjing University of Posts and Telecommunications, Nanjing, China; 3 Shanghai Institute of Liver Diseases, Shanghai, China; 4 Key Laboratory of Bioanalysis and Metrology for State Market Regulation, Shanghai Institute of Measurement and Testing Technology, Shanghai, China

**Keywords:** Hepatitis B virus, RNase-H assisted target recycling amplification, Toehold-mediated strand displacement, DNA three-way junction, rcDNA

## Abstract

Chronic hepatitis B virus (HBV) infection remains a significant global health challenge. Accurate quantification of HBV relaxed circular DNA (rcDNA) is critical for monitoring viral replication and assessing disease progression. However, conventional isothermal amplification methods are often compromised by limited specificity. To address these limitations, we engineered a toehold-triggered switchable DNA three-way junction (3WJ) protective nanoprobe that synergizes RNase H-assisted target recycling amplification (RATRA) with toehold-mediated strand displacement (TMSD). Leveraging the programmability of DNA nanotechnology, we constructed a rigid 3WJ scaffold featuring a “protective” nanoscale cavity at the branching point. This architecture effectively sequesters the ribonucleotides via steric hindrance, preventing non-specific enzymatic cleavage in the absence of the target. Upon specific recognition of the HBV rcDNA gap region, the nanoprobe undergoes a precise conformational switch, initiating a cyclic cleavage and signaling cascade. Governed by a dual-verification mechanism, the assay demonstrates exceptional specificity, enabling the discrimination of single-base mutations. Under optimized conditions, the method achieved a limit of detection (LOD) of 87.28 fM with a broad linear dynamic range. Validation in clinical serum samples revealed strong concordance with gold-standard quantitative real-time PCR (qRT-PCR). Consequently, this work presents a robust, modular, and cost-effective platform for point-of-care viral diagnostics.

**Graphical Abstract:**

**Figure:**
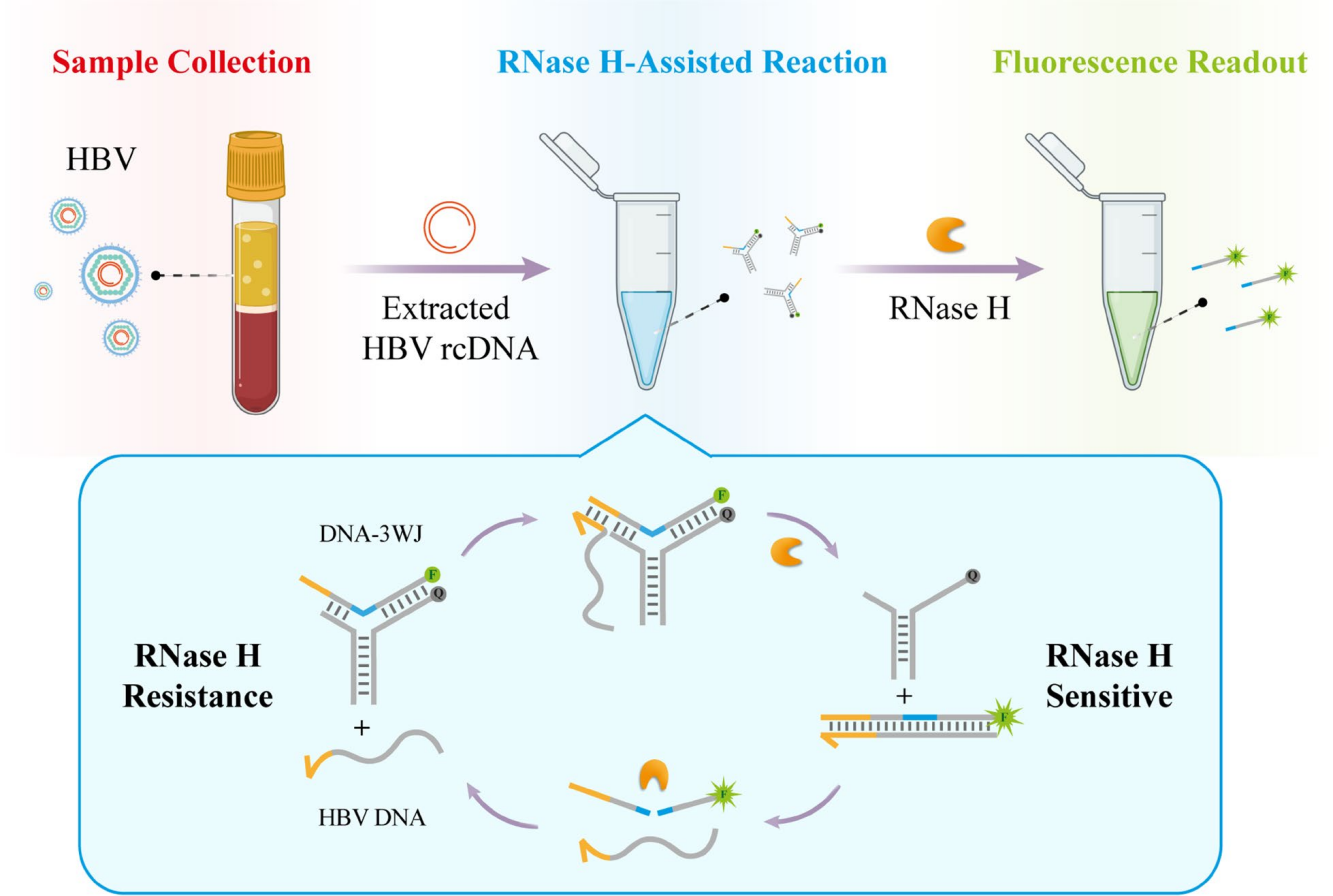
A DNA three-way junction nanoprobe undergoes toehold-triggered structural switching that exposes a hidden RNA/DNA duplex for RNase H cleavage, enabling highly specific cyclic detection of HBV rcDNA

**Supplementary Information:**

The online version contains supplementary material available at 10.1186/s12951-026-04344-y.

## Introduction

Hepatitis B virus (HBV) infection remains a serious global health challenge to this day, although safe and effective vaccines have been available since 1982 to prevent its spread [1]. According to the most recent World Health Organization statistics, as of 2022, approximately 254 million people worldwide suffer from chronic HBV infection [2, 3]. Alarmingly, an estimated 1.2 million new cases emerge, and around 1.1 million deaths occur annually largely due to complications such as cirrhosis and hepatocellular carcinoma (HCC) [4–6]. The level of HBV replication activity, as indicated by the viral load, provides valuable insights into disease progression, treatment efficacy, and the risk of developing liver complications [7, 8]. Consequently, accurately measuring and monitoring HBV viral load is crucial for optimizing patient care, implementing timely interventions, and reducing the global burden of HBV infection-related complications.

The circulating HBV DNA predominantly exists as relaxed circular DNA (rcDNA) [9, 10]. Detection of rcDNA provides a direct measure of active viral replication and offers a more precise assessment of viral dynamics compared to conventional serological markers. The rcDNA is a 3.2 kb partially double-stranded viral genome with a unique structure comprising a full-length negative strand and a shorter, incomplete positive strand, resulting in distinct single-stranded gap regions [11]. From a diagnostic perspective, these structural features make HBV rcDNA ideal for nuclease-assisted isothermal amplification. Traditional PCR requires precise thermal cycling, expensive equipment, and denaturation to separate double-stranded DNA. In contrast, molecular probes in isothermal assays can directly recognize accessible gap regions of rcDNA at a constant temperature, which improves assay portability and cost-effectiveness. It is therefore well-suited for measuring viral loads, especially in resource-limited settings. Compared to popular isothermal methods like LAMP and CRISPR-based diagnostics, direct, single-step nuclease-assisted target-recycling offers clear advantages. These benefits include simpler design and better kinetic efficiency. For example, LAMP is highly sensitive but often needs complex primer sets and multistep amplification, which makes it prone to nonspecific products [12, 13]. Similarly, CRISPR-based diagnostic methods, though powerful in their programmable specificity, typically necessitate a pre-amplification step (such as RPA or PCR) and trans-cleavage activation to achieve adequate sensitivity [14–17]. This requirement for multi-enzyme coordination not only increases the risk of aerosol contamination but also limits their deployment in resource-constrained settings. In contrast, RNase H-assisted target recycling amplification (RATRA) provides a more direct and efficient alternative [18, 19]. This method directly initiate multiple cycles of probe hybridization and cleavage without the need for intricate probe sets and multi-enzyme coordination. The direct recycling mechanism ensures a high turnover rate and robustness, yielding more controlled amplification. Despite these advantages, the practical application of conventional RNase H-assisted assays compromised by the relatively poor specificity. This arises from the fact that RNase H recognizes and cleaves DNA–RNA hybrids in a sequence-independent manner, making the system highly sensitive to partial or mismatched hybridization events [20–22]. Additionally, the heightened mutational variability of HBV, stemming from the lack of proofreading activity in its polymerase, puts forward greater demands on the reliablity of detection methods. To tackle this challenge, a kinetic approach named toehold-mediated strand displacement (TMSD) has stand out for programable design of nucleic acid probes, enabling improved specificity by minimizing the likelihood of non-specific binding of probes [23, 24]. By incorporating such structural features, the risk of non-specific amplification can be effectively mitigated, ultimately enhancing assay specificity and reliability. Due to the fact that RNase H cleaves the RNA strand within a DNA-RNA heteroduplex in a sequence-independent manner, it is temporarily difficult to be implemented in probes that use RNase H for signal amplification.

**Scheme 1:**
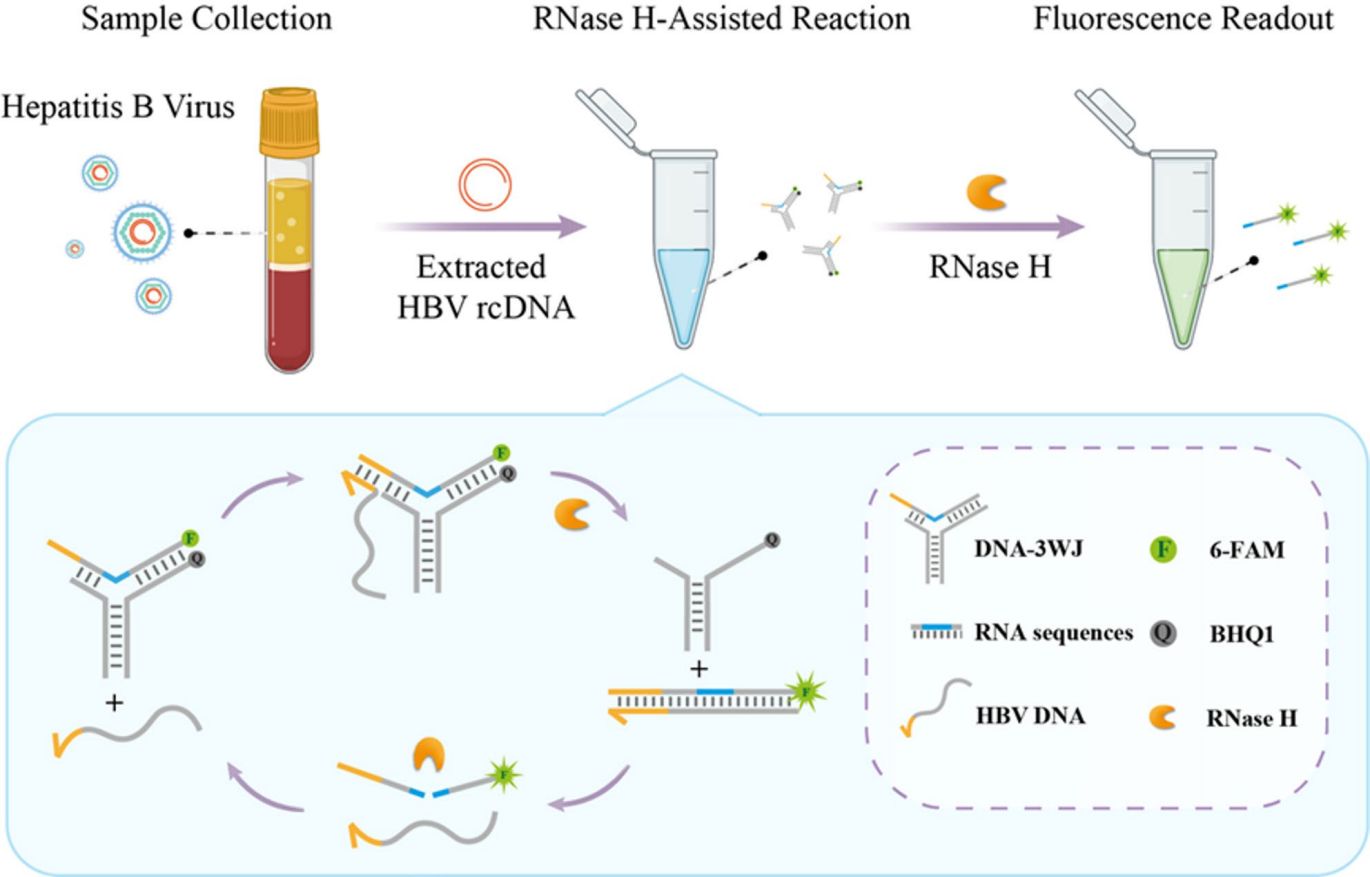
Schematic illustration of toehold-triggered switchable DNA three-way junction nanoprobe for highly specific RNase H-assisted HBV rcDNA detection

With the rapid evolution of structural DNA nanotechnology, an extensive repertoire of sophisticated DNA nanostructures has been meticulously engineered, largely enabled by the unique ability of DNA strands to branch into diverse junction architectures. These nanostructures have been successfully deployed across numerous biomedical applications, ranging from targeted drug delivery to advanced biosensing, due to their exceptional programmability and biocompatibility [25–30]. In nature and synthetic design, various junction motifs, especially multi-arm junctions stand out as fundamental scaffolds that drive this versatility. Among these structural cornerstones, the Three-Way Junction (3WJ) stands out as a particularly versatile building block. Characterized by three double-helical arms radiating from a central branch point, the 3WJ motif provides a unique balance of structural rigidity and conformational flexibility, making it an ideal candidate for developing dynamic, switchable nanoprobes. The 3WJ is a unique branching structure consisting of three double-helical arms connected at the branching point [31, 32]. It has been shown that DNA-3WJ with fully base pairing forms only an open, non-stacked conformation, i.e., a non-planar Y-shaped structure [31, 33], creating a nanoscale cavity due to complementary base unpairing at and near its branching point [34]. Owing to the cleavage property of RNase H that mismatches at the cleavage site can significantly impair its cleavage activity [35, 36], this nanoscale cavity in DNA-3WJ structure offers a viable approach to enhance the specificity of RNase H-mediated cleavage. Herein, we have developed an evolved toehold-bearing nucleic acid probe, namely toehold-triggered switchable DNA 3WJ Protective Nanoprobe. This design strategically integrates the high-specificity recognition of a toehold-mediated mechanism with a unique “protective” 3WJ architecture, which effectively sequesters the probe from non-specific enzymatic cleavage. Upon precise recognition of the HBV rcDNA gap region, the probe undergoes a structural switching event that initiates RNase H-assisted target recycling (Scheme 1). By synergizing the precision of structural switching with the catalytic efficiency of RNase H, this strategy achieves highly specific and sensitive quantification of HBV rcDNA, providing a robust tool for the accurate assessment of viral loads in clinical diagnostics.

## Experimental section

### Materials and reagents

RNase H was purchased from New England Biolabs (Beijing) Co., Ltd. Bovine serum albumin (BSA) was obtained from Merck Chemical Technology Co., Ltd. Human serum albumin (HSA) was supplied by Nanjing Fengbo Biotechnology Co., Ltd. Magnesium chloride (MgCl_2_), Tris(hydroxymethyl)aminomethane (Tris), boric acid (H_3_BO_3_), disodium ethylenediaminetetraacetate dihydrate (Na_2_EDTA·2H_2_O), magnesium acetate tetrahydrate (Mg(CH_3_COO)_2_·4H_2_O), and sodium chloride (NaCl) were all purchased from Shanghai Sinopharm Chemical Reagent Co., Ltd. Ammonium persulfate (APS), N,N, N’,N’-tetramethylethylenediamine (TEMED), Tris-HCl solution, diethyl pyrocarbonate (DEPC)-treated water, 30% acrylamide/bis-acrylamide solution (19:1), DNA molecular weight marker, and 4 S Gel-Red nucleic acid stain were obtained from Sangon Biotech (Shanghai) Co., Ltd. All other chemical reagents were of analytical grade and used without further purification. Ultrapure water (> 18 MΩ·cm) obtained from a Milipore water purification system was used for all solution preparations. The DNA sequences used in the experiments were synthesized by Sangon Biotech (Shanghai) Co., Ltd., and their details are listed in Table S1.

### Apparatus

The melting temperature of the DNA-3WJ structure was determined by a CFX Opus 96 real-time quantitative PCR instrument (Bio-Rad, USA). All the enzymatic reactions were conducted at the optimal temperature in a Veriti 96-Well Thermal Cycler PCR instrument (Thermo Fisher Scientific, China). The fluorescence emission spectra were recorded by a FluoroMax Plus fluorescence spectrophotometer (HORIBA Scientific, USA). A G: BOX F3 gel imager (Syngene, UK) was employed to visualize and image the polyacrylamide gel after staining in the PAGE verification experiment.

### Preparation of DNA-3WJ

**Fig. 1:**
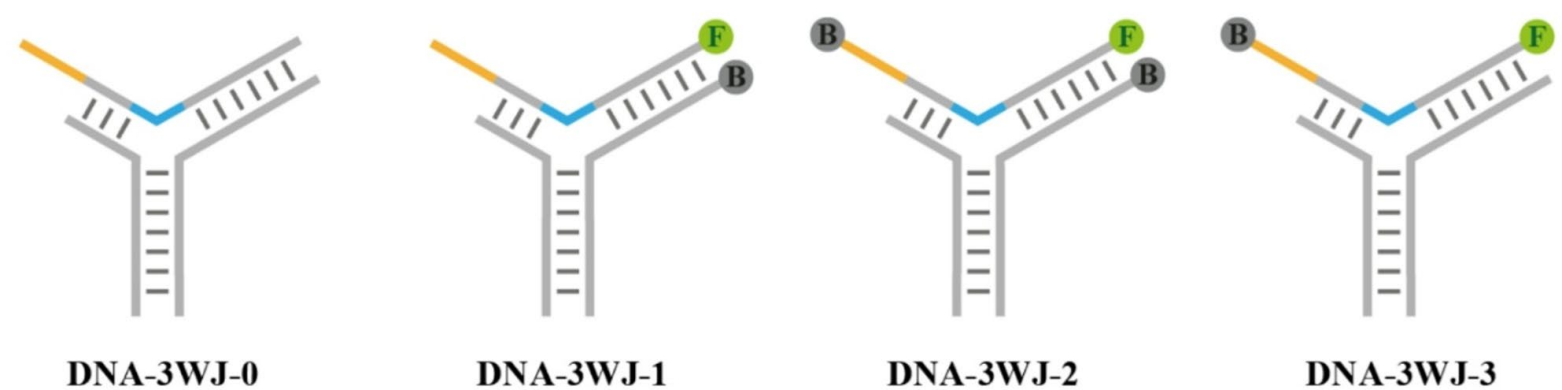
Naming convention for DNA-3WJ protective nanoprobes

First, the purchased single-stranded DNA (ssDNA) was dissolved in an appropriate amount of ultrapure water to an initial concentration of 100 µM. Subsequently, 15 µL of Probe 1 (P1, P1-Q), 10 µL of Probe 2 (P2), and 10 µL of template strand (Template, Template-F, or Template-FQ) were mixed at a molar ratio of 1.5:1:1, under the buffering conditions of 1x TM buffer and the total volume of 100 µL. The mixture was incubated at 95 °C for 5 min, then annealed from 95 °C to 25 °C at a rate of -0.1 °C/6 s to form DNA-3WJ structures. The naming convention for DNA-3WJ structures composed of different P1 or template-F was shown in the Fig. 1.

### HBV DNA detection by the RATRA and TMSD assisted DNA-3WJ protective nanoprobe assay

52 µL 1×TM buffer, 2 µL 0.3% BSA, and 2 µL 0.9 µM DNA-3WJ-1 were mixed first. Then, 2 µL (5 U) RNase H was added to the experimental group (with 2 µL ultrapure water to reach 60 µL). The control group received 4 µL ultrapure water instead of RNase H, keeping the final volume consistent. After incubation at 37 °C for 30 min, the fluorescence intensity of the sample was measured using a fluorescence spectrophotometer at excitation/emission = 495/520 nm. The limit of detection (LOD) was calculated using the 3σ blank method, with the background standard deviation derived from RNase H-containing blanks.

### Polyacrylamide gel electrophoresis (PAGE) verification

To further confirm the feasibility of the experimental protocol, polyacrylamide gel electrophoresis (PAGE) was performed. Considering the potential interference of the quenching group (BHQ1) on Gel-Red staining, unmodified DNA strands were exclusively used in all electrophoresis experiments. The PAGE analysis was conducted using a 10% polyacrylamide gel. Prior to loading, each DNA sample was mixed with 6×loading buffer at a volume ratio of 5:1. Electrophoresis was carried out at a constant voltage of 80 V for 90 min in 1×TBE running buffer at room temperature. After electrophoresis, the gel was stained with 4 S Gel-Red nucleic acid stain (diluted 1:10,000 in ultrapure water) for 20 min in the dark. Finally, the stained gel was visualized and imaged using a G: BOX F3 gel imager under UV transillumination (302 nm).

### Clinical sample analysis

Serum samples were obtained from Zhongshan Hospital Affiliated to Fudan University. Venous blood from all study participants was collected using SST separation gel and coagulant (Becton Dickinson, USA). Following centrifugation at 3000 rpm for 10 min, serum specimens were harvested and stored at -80℃ until utilization. This research was approved by the Ethics Committee of Zhongshan Hospital Affiliated to Fudan University (B2022-482R2), and all patients provided signed informed consent. Detailed information of all samples in this work were listed in Table S2.

## Results and discussion

### Definition and feasibility verification of DNA-3WJ protective nanoprobe

The DNA-3WJ Protective Nanoprobe was engineered using three specific oligonucleotides: P1, P2, and a Template strand containing four ribonucleotide inserts. To facilitate target recognition, a toehold domain was strategically positioned at the 5’ end of the P1/Template duplex. Notably, the ribonucleotides are located at the 3WJ branching point, where the presence of unpaired complementary bases and the unique junction architecture create a nanoscale cavity. This structure exerts steric hindrance that protects the RNA bases from non-specific RNase H cleavage in the absence of the target.

**Fig. 2:**
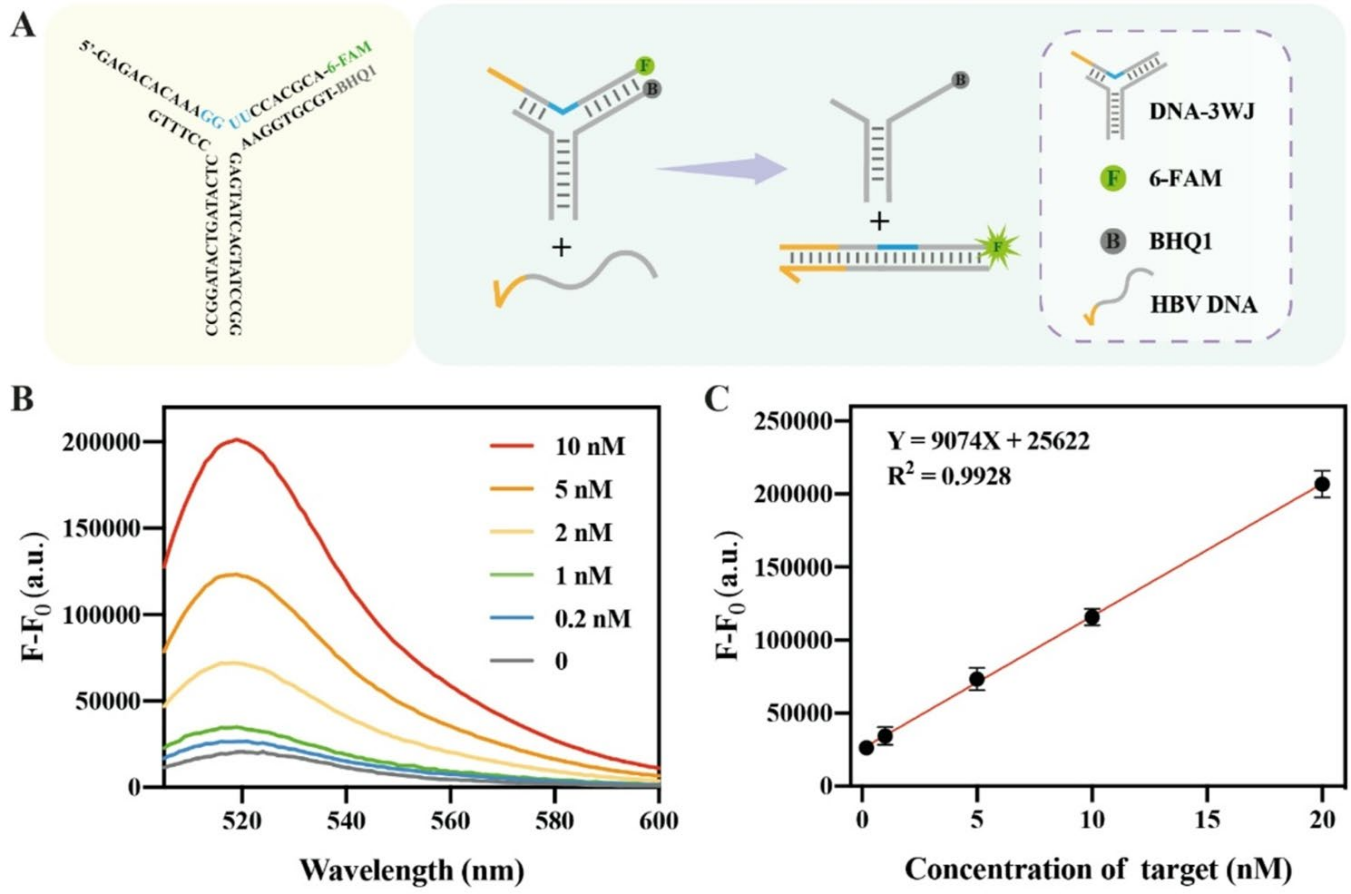
Feasibility of Strand Displacement Ability of DNA-3WJ Protective Nanoprobe. (**A**) Schematic illustration of the DNA-3WJ Protective Nanoprobe. (**B**) Signal-to-noise ratio of different R probes. (**C**) Calibration curve for HBV DNA detection without RATRA. The concentration of DNA-3WJ is 150 nM, with presence of 5 U RNase H. All DNA components were mixed and incubated for 30 min before measurement. The error bars represent the standard deviation (SD) across three repetitive experiments

The sensing mechanism is initiated only upon the introduction of the target HBV rcDNA. The target binds to the toehold, triggering TMSD, which releases the Template strand from the 3WJ scaffold. Once liberated, the Template strand forms a fully complementary DNA/RNA heteroduplex with the target, rendering it susceptible to RNase H cleavage. This enzymatic digestion releases the target back into the solution to initiate subsequent rounds of target recycling, thereby amplifying the fluorescent signal.

Based on this principle, we first constructed the DNA-3WJ Protective Nanoprobe, which comprises three oligonucleotide strands (P1, P2, and Template), with RNA bases strategically positioned at the junction to form a nanoscale cavity resistant to RNase H. A toehold domain is engineered at the extension of the double helix formed by the hybridization of the Template and P1 strands. Subsequently, the probe’s response to target-induced TMSD was evaluated using gradient concentrations of HBV DNA, as illustrated in the probe design schematic presented in Fig. 2A. As the target concentration increased, progressive displacement of the Template strand led to a corresponding fluorescence enhancement (Fig. 2B), showing a strong linear correlation between fluorescence intensity and HBV DNA concentration (Y = 9074X + 25622, R² = 0.9928; Fig. 2C). The limit of detection was determined to be 36.37 pM.

**Fig. 3:**
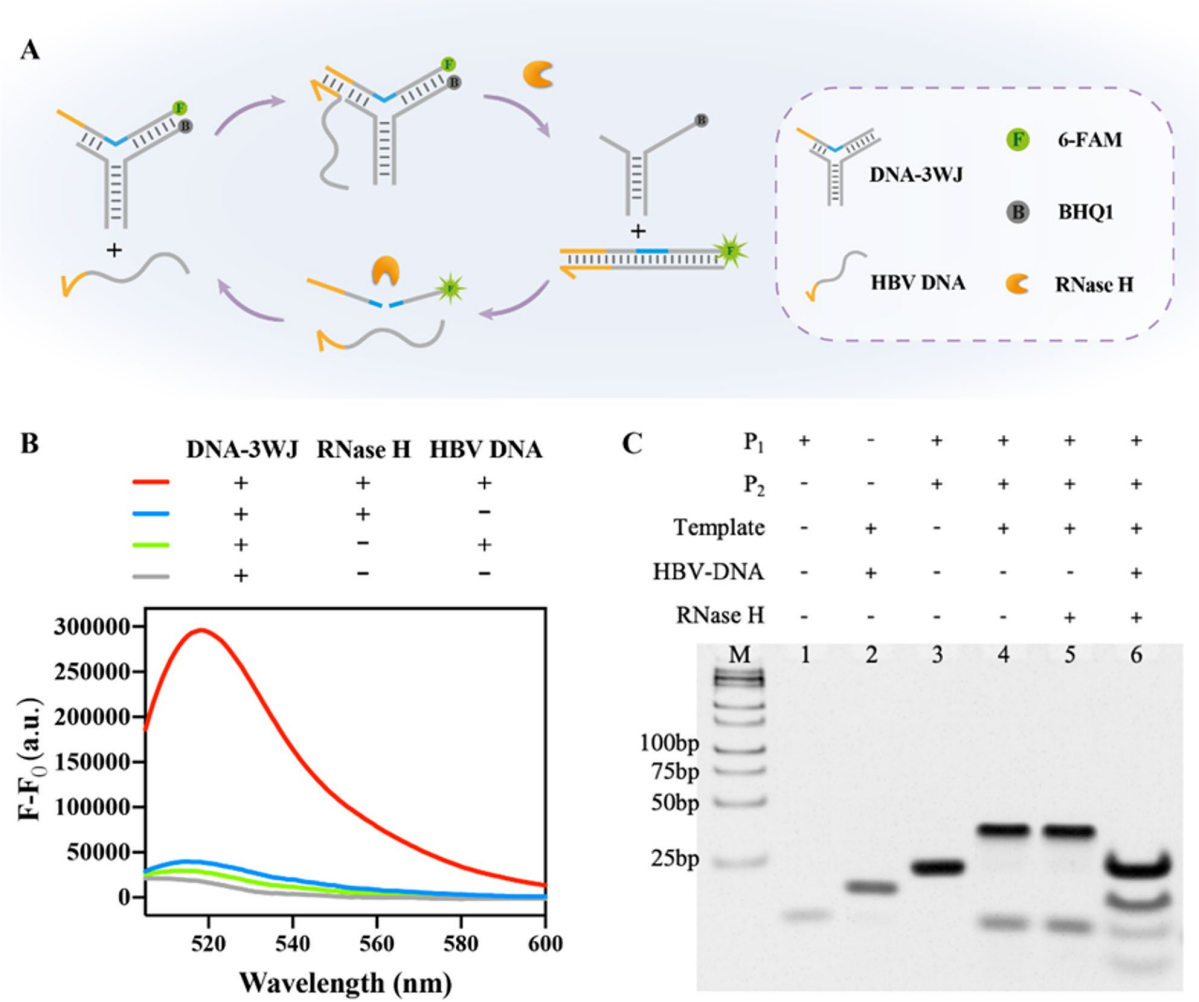
Feasibility of the RATRA and TMSD assisted DNA-3WJ Protective Nanoprobe Assay. (**A**) Schematic illustration of RNase H-assisted amplification by DNA-3WJ Protective Nanoprobe. Fluorescent spectrogram (**B**), and native PAGE result (**C**) of each group. In fluorescent testing, the concentration of DNA-3WJ is 150 nM, while the concentration of target HBV DNA is 20 nM, with the presence/absence of 5 units RNase H. The concentration of all nucleic acid components was increased by five times for native PAGE. All DNA components were mixed and incubated for 30 min before measurement

Building on this foundation, we further explored the feasibility of the RNase H-driven DNA-3WJ Protective Nanoprobe for target recycling and signal amplification via TMSD. Specifically, HBV DNA hybridizes with the Template strand through TMSD to form double-stranded DNA, which serves as a substrate for RNase H. Following cleavage of the Template strand, HBV DNA is released back into the reaction system to participate in subsequent cycles of strand displacement and cleavage, thereby enabling signal amplification (Fig. 3A). Fluorescence intensity measurements revealed that the DNA-3WJ Protective Nanoprobe alone or when DNA-3WJ and HBV DNA were present simultaneously exhibited negligible fluorescence (Fig. 3B, grey curve and green curve), and RNase H induced minimal non-specific cleavage of the probe (Fig. 3B, blue curve). In contrast, the presence of HBV DNA facilitated repeated entry into the reaction cycle, mediating continuous displacement and cleavage of the Template strand, which resulted in a robust fluorescence signal (Fig. 3B, red curve). PAGE results further validated that Template strand displacement and cleavage occurred exclusively in the presence of HBV DNA (Fig. 3C).

### Optimization of experimental conditions for the RATRA and TMSD assisted DNA-3WJ protective nanoprobe assay

**Fig. 4:**
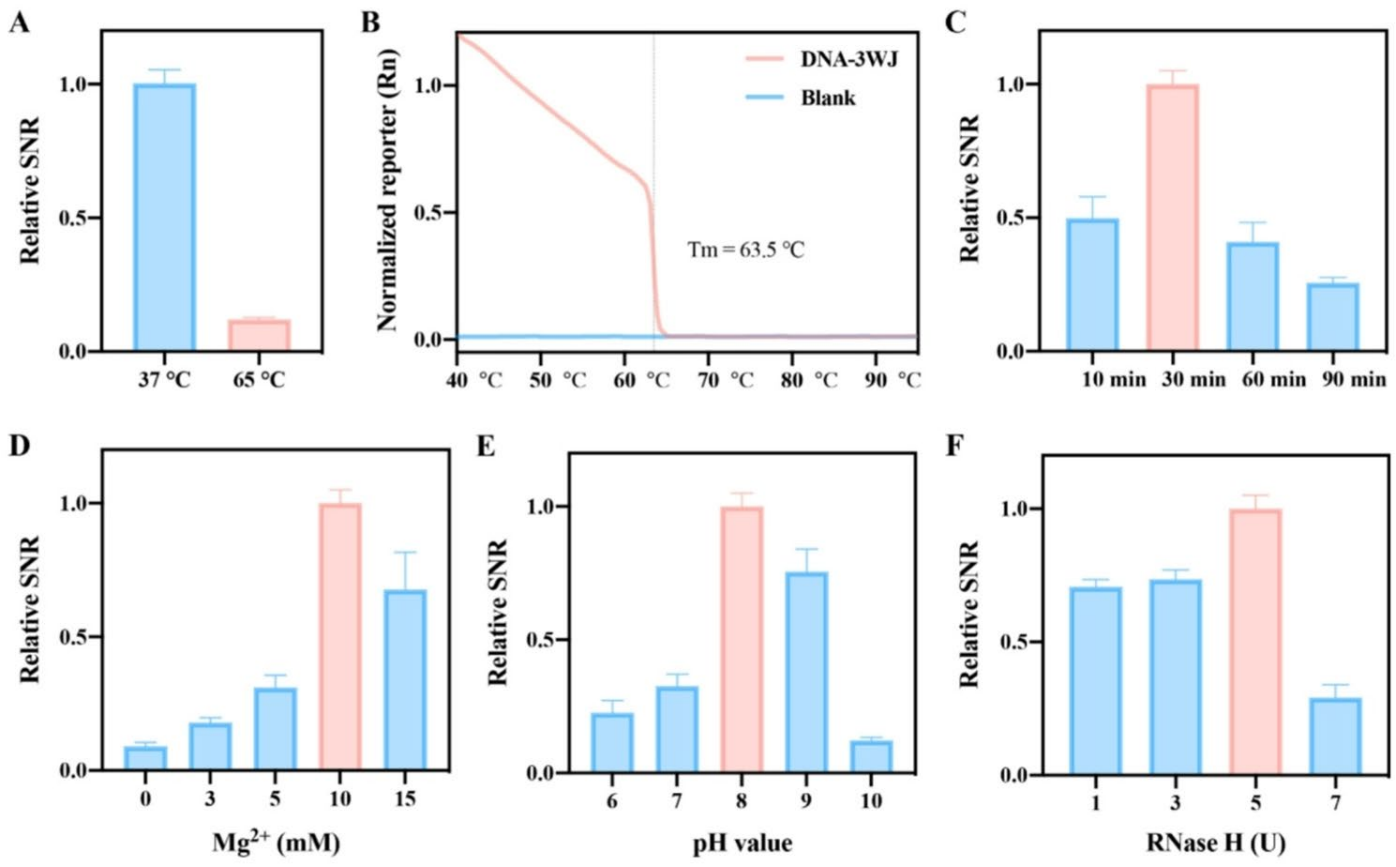
Optimization of the reaction conditions with the relative SNR serving as the guiding indicator: (**A**) Reaction temperature, (**B**) melt curve of DNA-3WJ, (**C**) reaction time, (**D**) concentration of Mg^2+^, (**E**) pH value, and (**F**) the amount of RNase H. The concentration of probe P-R and F is 150 nM, while the concentration of target HBV DNA is 5 nM, with presence of 5 units RNase H. The error bars represent the standard deviation (SD) across three repetitive experiments

To enhance detection efficiency, we optimized key experimental conditions, including reaction temperature, reaction time, buffer magnesium ion concentration, pH value, and RNase H dosage in the system. Results were presented as relative signal-to-noise ratio (SNR), calculated using the formula: SNR = (F_T_ - F_0_)/(F_R_ - F_0_), where F_0_ denotes the fluorescence intensity of DNA-3WJ alone, FR represents that of DNA-3WJ mixed with RNase H, and FT is the intensity of the mixture containing DNA-3WJ, RNase H, and HBV-DNA. Optimization was performed with a reference target concentration of 5 nM. Commercial RNase H exhibits two optimal reaction temperatures (37 ℃ and 65 ℃). Separate SNR tests for each temperature revealed that the SNR at 65 °C was only 12.04% of that at 37 ℃ (Fig. 4A). To elucidate this phenomenon, we measured the melting curve of the DNA-3WJ Protective Nanoprobe, which yielded a Tm value of ~ 63.5 ℃ (Fig. 4B). Thus, the significant SNR reduction at 65 ℃ may result from probe dissociation. With prolonged reaction time, the relative SNR first increased and then decreased, peaking at 30 min (Fig. 4C). This observation might be attributed to the elevated proportion of non-specific reactions over time. Therefore, the optimal reaction duration was determined to be 30 min. Mg^2+^ concentration and pH value are critical for DNA-3WJ assembly, subsequent TMSD reactions, and RNase H catalytic activity. As shown in Fig. 4D, the relative SNR first increased and then decreased with increasing Mg^2+^ concentration, reaching a maximum at 10 mM. This concentration was designated as the optimal magnesium ion level. The optimal pH value was identified as 8 (Fig. 4E). Finally, we optimized the RNase H concentration in the system. As illustrated in Fig. 4F, the SNR reached its maximum at an RNase H concentration of 5 U. This dosage was selected as the optimal concentration for subsequent experiments.

### Specificity of the RATRA and TMSD assisted DNA-3WJ protective nanoprobe assay

**Fig. 5:**
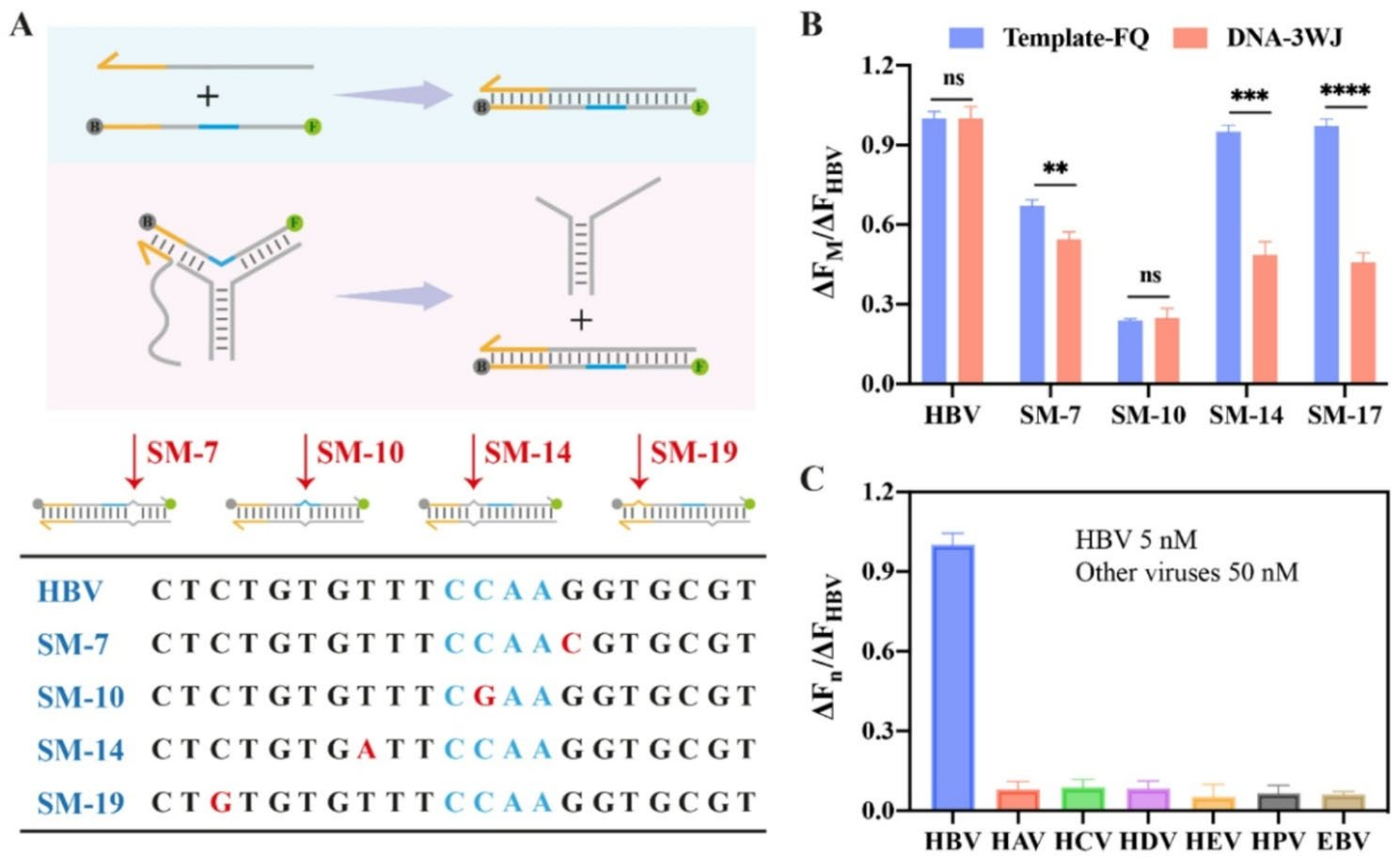
Specificity of the RATRA and TMSD assisted DNA-3WJ Protective Nanoprobe Assay. (**A**) Sequences of targets mismatched in the RATRA and TMSD assisted DNA-3WJ Protective Nanoprobe Assay. (**B**) The role of the RATRA and TMSD assisted DNA-3WJ Protective Nanoprobe Assay in enhancing the ability to distinguish single base mismatches. (**C**) Nucleic acids of other viruses with the 10 folds titer were assayed negatively. The concentration of probe P-R and F is 150 nM, while the concentration of target HBV DNA is 5 nM, with presence of 5 units RNase H. The error bars represent the standard deviation (SD) across three repetitive experiments

Distinguishing single-base mutations and highly homologous HBV DNAs remains a significant challenge in clinical diagnostics. The specificity of our system is governed by a dual-verification mechanism: first, mismatches near the toehold region significantly impede the TMSD kinetics; second, any sequence variations near the ribonucleotide site disrupt the efficiency of RNase H-mediated cleavage. This dual-gate architecture ensures high-fidelity discrimination of HBV rcDNA under isothermal conditions. Thus, we evaluated the system using DNA strands similar to wild-type HBV DNA but harboring a single-base mutation at different positions (Fig. 5A). In order to compare the difference between the DNA-3WJ structure and a single Template strand, we used template strands modified at both ends for this experiment. The corresponding DNA-3WJ Protective Nanoprobe is named DNA-3WJ-3. As shown in Fig. 5B, compared with the single-stranded probe, the DNA-3WJ Protective Nanoprobe exhibits improved detection specificity. Although the specificities of the two probes are almost identical when the mutation site is located at the RNase H cleavage site, the ratio of the relative fluorescence intensity of the mutant target to the wild-type target is improved from 0.67 to 0.54 when the single-nucleotide mutation is adjacent to the RNase H cleavage site. When the single-nucleotide mutation is 2 bases away from the RNase H cleavage site, this ratio is enhanced from 0.94 to 0.48. Additionally, when the single-nucleotide mutation is located within the toehold region, the ratio is improved from 0.97 to 0.46. These results demonstrate that the DNA-3WJ Protective Nanoprobe can enhance the recognition capability for single-nucleotide mutations at non-RNase H cleavage sites, particularly those distant from the cleavage site. To further evaluate the specificity of the proposed strategy, we used nucleic acids from other hepatitis viruses or other DNA viruses at a 10-fold higher concentration as controls. Fluorescence signals similar to those of the blank control were observed (Fig. 5C).

### Quantitative performance of the RATRA and TMSD assisted DNA-3WJ protective nanoprobe assay

**Fig. 6:**
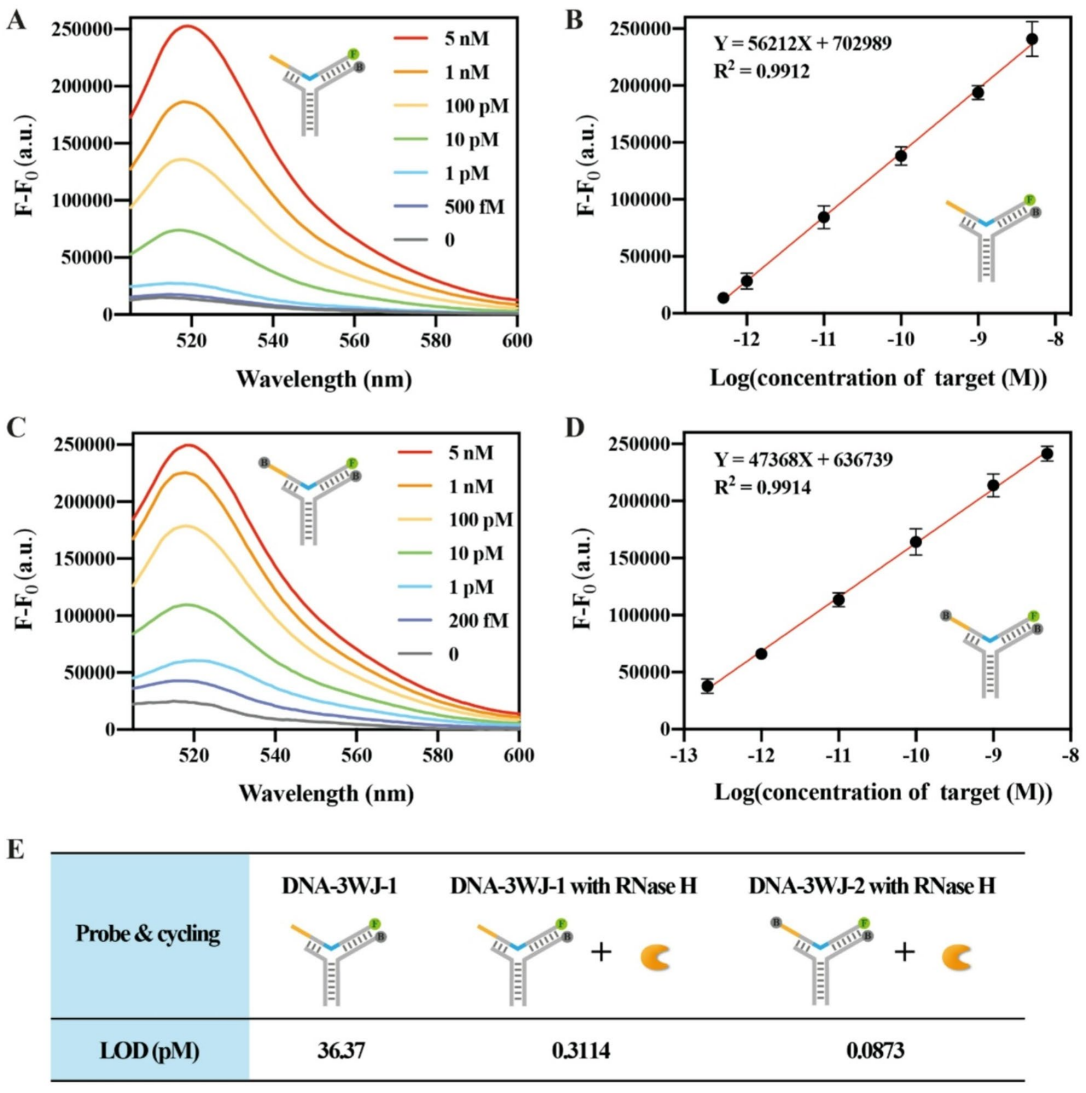
Sensitivity of the RATRA and TMSD assisted DNA-3WJ Protective Nanoprobe Assay. (**A**) Fluorescence emission spectrum of the assay with DNA-3WJ-1 upon addition of different concentrations of HBV DNA (from bottom to top): 0, 500 fM, 1 pM, 10 pM, 100 pM, 1 nM, and 5 nM, with the proposed method. (**B**) Calibration curve for HBV DNA detection with DNA-3WJ-1. (**C**) Fluorescence emission spectrum of the assay with DNA-3WJ-2 upon addition of different concentrations of HBV DNA (from bottom to top): 0, 200 fM, 1 pM, 10 pM, 100 pM, 1 nM, and 5 nM, with the proposed method. (**D**) Calibration curve for HBV DNA detection with DNA-3WJ-2. The error bars represent the standard deviation (SD) across three repetitive experiments

A series of samples containing different concentrations of HBV DNA ranging from 500 fM to 5 nM were investigated under the optimized condition for limit of detection (LOD) measurement. The fluorescence intensity gradually increased with the increasing HBV DNA concentration. The fluorescence spectrums for the increasing concentration of target were shown in Fig. 6A. The calibration curve was depicted in Fig. 6B, with a corresponding linear equation of Y = 56212X + 702,989 (R square = 0.9912) (X represents the logarithm of HBV DNA concentration). The LOD was evaluated at 311.35 fM. The sensitivity of the proposed method has been significantly improved compared to that without RATRA. Moreover, it is either comparable to or superior to that of previously reported assay based on DNA circuit for single stranded DNA detection (Table S3).

To further improve detection sensitivity, we designed the Template strand into a structure similar to TaqMan probes. Specifically, a second BHQ group was introduced at the opposite end of the 6-FAM moiety, forming the probe DNA-3WJ-2 together with P1-Q and P2. A series of samples containing HBV DNA at concentrations ranging from 200 fM to 5 nM were analyzed using DNA-3WJ-2. The obtained fluorescence spectra are shown in Fig. 6C. The calibration curve is depicted in Fig. 6D, with the corresponding linear equation Y = 47368X + 636,739 (R² = 0.9914), where X represents the logarithm of the HBV DNA concentration. The limit of detection (LOD) was calculated to be 87.28 fM. The additional BHQ group contributes to improvements in detection sensitivity and a lower LOD. Under identical reaction substrates and conditions, DNA-3WJ-2 exhibited lower background fluorescence due to the presence of an additional quencher. For DNA-3WJ-1, the fluorescence intensity of the 500 fM target group shows only a minor difference from the target-free background group, meaning the background signal is high and the specific signal triggered by the target is severely masked. In sharp contrast, with DNA-3WJ-2, even at a lower target concentration (200 fM) the fluorescence intensity of the target group exhibits a markedly larger difference from the target-free background group. Upon initiation of cyclic amplification, the short 6-FAM labeled oligonucleotides are released into the system, and all quenchers lose their quenching effect as a result of increased spatial separation. After complete reaction of all probes, the fluorescence intensities of both probe systems eventually converge. The additional BHQ group enhances detection sensitivity and reduces the LOD.

The LODs obtained from the three different fluorescent probes and cycling modes involved in this manuscript are shown in Fig. 6E. Under identical reaction substrates and conditions, DNA-3WJ-2 exhibits lower background fluorescence due to the extra quencher, which contributes to improving the SNR of the reaction. Therefore, DNA-3WJ-2 was selected as the probe for subsequent detection.

**Fig. 7:**
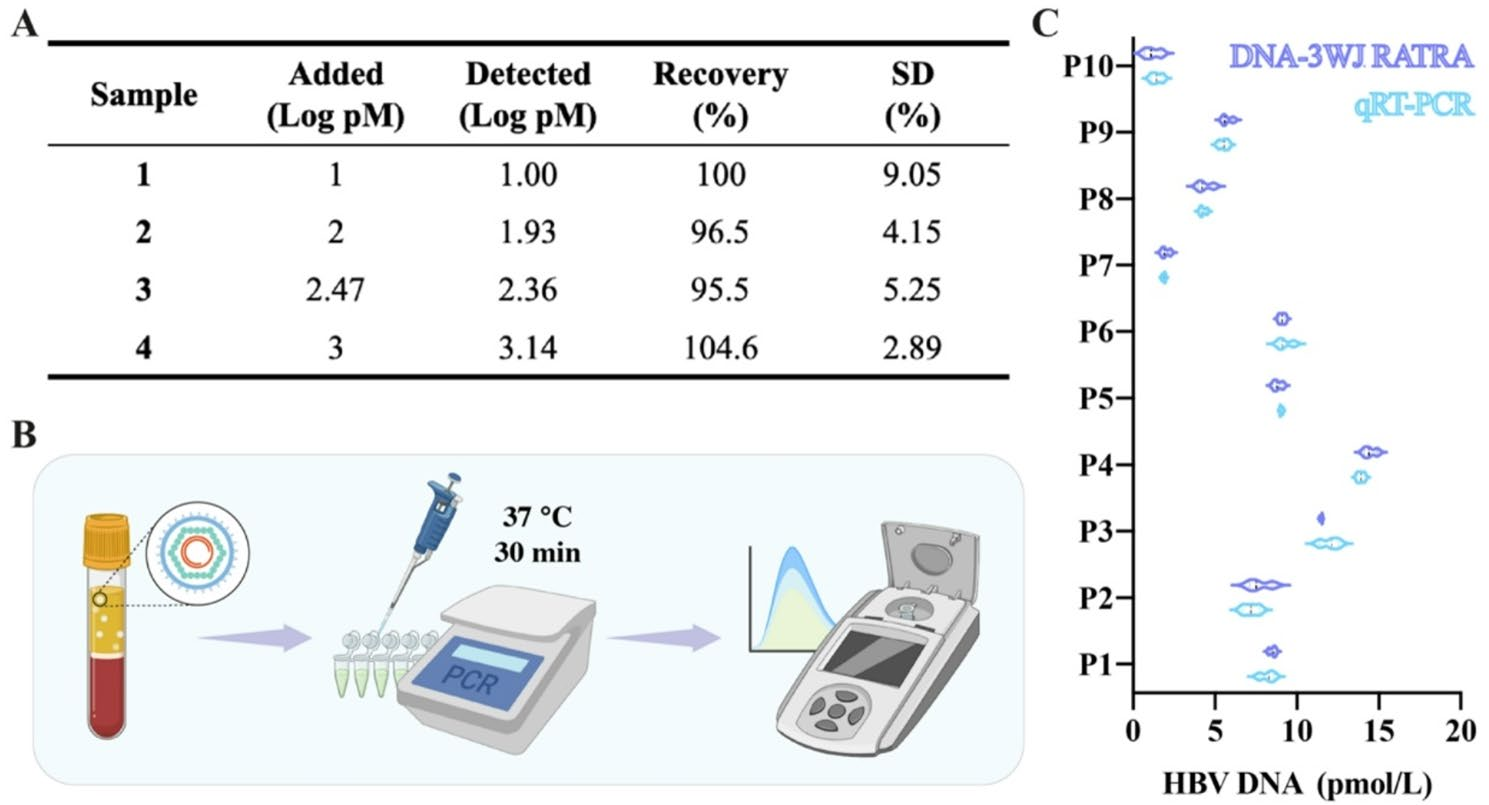
RATRA and TMSD assisted DNA-3WJ Protective Nanoprobe assay for HBV rcDNA in clinical samples. (**A**) Recoveries of HBV DNA in the human serum samples (*N* = 3). (**B**) Schematic illustration of the HBV DNA detection workflow. (**C**) HBV DNA detection in 10 clinical serum samples using DNA-3WJ Protective Nanoprobe Assay and TMSD assisted DNA-3WJ Protective Nanoprobe assay compared with qRT-PCR. The error bars represent the standard deviation (SD) across three repetitive experiments

As blood transmission is the primary route of HBV spread, detecting HBV DNA in blood is critical for diagnosing HBV infection, monitoring treatment progress, and preventing further transmission. To assess its performance, we performed recovery experiments in 10-fold diluted healthy human serum spiked with 10 pM, 100 pM, 300 pM and 1 nM HBV DNA, respectively. Figure 7A shows the serum detection results are highly close to those in standard buffer, with fluorescence-based recoveries of 95.5–104.6% for spiked serum samples. Furthermore, we explored the applicability of the RATRA and TMSD assisted DNA-3WJ Protective Nanoprobe Assay in clinical HBV diagnosis (assay procedure in Fig. 7B). This study enrolled patients with serum HBV DNA levels near the 2000 IU/mL (about 0.96pmol/L) therapeutic threshold, as this cutoff represents a key decision point for antiviral therapy and allows for the evaluation of diagnostic performance in clinically relevant scenarios. Using this strategy, we analyzed 10 serum samples from HBV DNA-positive patients and compared the results with qRT-PCR quantification (Fig. 7C), and there was no statistical diference between the DNA-3WJ Protective Nanoprobe Assay and RT-qPCR. The current study was conducted with a relatively small panel of clinical samples. However, the limited sample size did not compromise the reliability of the key observations, as all samples were tested rigorously and demonstrated consistent performance. These experiments confirm the proposed method has sensitivity and accuracy for HBV detection, exhibiting great potential in clinical monitoring.

## Conclusion

In summary, we have successfully developed a highly specific and sensitive fluorescence sensor for HBV rcDNA detection by synergizing RATRA with a toehold-triggered switchable DNA-3WJ Protective Nanoprobe. This study addresses a fundamental bottleneck in RNase H-assisted assays—background leakage—by exploiting the conformational control of DNA nanostructures. The unique 3WJ architecture creates a protective nanoscale cavity that shields the substrate from non-specific cleavage, a protection mechanism that is conditionally released only upon specific toehold-triggered strand displacement. This design not only retains the high sensitivity of enzymatic cycling (with a detection limit of 87.28 fM) but also significantly enhances specificity, enabling precise discrimination of single-base mismatches—a capability that is lacking in conventional RATRA methods. The assay’s excellent performance in complex serum matrices and its consistency with gold-standard qRT-PCR confirm its clinical viability. Furthermore, its modular design offers a versatile foundation for developing “smart” diagnostic devices adapted to other targets. By synergizing the respective strengths of structural and dynamic DNA nanotechnology, this work underscores the transformative potential of programmable DNA nanostructures in propelling the advancement of precise, point-of-care molecular diagnostics.

## Supplementary Information

Below is the link to the electronic supplementary material.

Supplementary Material 1.

## Data Availability

No datasets were generated or analysed during the current study.

## References

[1] Jeng WJ, Papatheodoridis GV, Lok ASF, Hepatitis B. Lancet. 2023;401(10381):1039–52. 36774930 10.1016/S0140-6736(22)01468-4

[2] Hsu Y-C, Huang DQ, Nguyen MH. Global burden of hepatitis B virus: current status, missed opportunities and a call for action. Nat Reviews Gastroenterol Hepatol. 2023;20(8):524–37. 10.1038/s41575-023-00760-9 37024566

[3] Zhang C, Liu Y, Zhao H, Wang G. Global Patterns and Trends in Total Burden of Hepatitis B from 1990 to 2019 and Predictions to 2030. Clin Epidemiol. 2022;14(null):1519–33. 36540899 10.2147/CLEP.S389853 PMC9760077

[4] Si J, Zou Y, Gao Y, Chen J, Jiang W, Shen X, Zhu C, Yao Q. tRF-3a-Pro: A Transfer RNA-Derived Small RNA as a Novel Biomarker for Diagnosis of Hepatitis B Virus-Related Hepatocellular Carcinoma. Cell Prolif. 2025;58(7):e70006. 39993386 10.1111/cpr.70006 PMC12240632

[5] Mao X, Cheung KS, Peng C, Mak LY, Cheng HM, Fung J, Peleg N, Leung HHW, Kumar R, Lee JH, Shlomai A, Yuen MF, Seto WK. Steatosis, HBV-related HCC, cirrhosis, and HBsAg seroclearance: a systematic review and meta-analysis. Hepatol. 2023;77(5):1735–45. 10.1002/hep.32792 36111362

[6] Campbell C, Wang T, McNaughton AL, Barnes E, Matthews PC. Risk factors for the development of hepatocellular carcinoma (HCC) in chronic hepatitis B virus (HBV) infection: a systematic review and meta-analysis. J Viral Hepatitis. 2021;28(3):493–507. 10.1111/jvh.13452 PMC8581992 33305479

[7] Meier M-A, Calabrese D, Suslov A, Terracciano LM, Heim MH, Wieland S. Ubiquitous expression of HBsAg from integrated HBV DNA in patients with low viral load. J Hepatol. 2021;75(4):840–7. 34004216 10.1016/j.jhep.2021.04.051

[8] Choi W-M, Yip TC-F, Wong GL-H, Kim WR, Yee LJ, Brooks-Rooney C, Curteis T, Clark LJ, Jafry Z, Chen C-H, Chen C-Y, Huang Y-H, Jin Y-J, Jun DW, Kim J-W, Park NH, Peng C-Y, Shin HP, Shin JW, Yang Y-H, Lim Y-S. Baseline Viral Load and On-Treatment Hepatocellular Carcinoma Risk in Chronic Hepatitis B: A Multinational Cohort Study. Clin Gastroenterol Hepatol. 2025;23(2):310–e3207. 39181430 10.1016/j.cgh.2024.07.031

[9] Tsukuda S, Watashi K. Hepatitis B virus biology and life cycle. Antiviral Res. 2020;182:104925. 32866519 10.1016/j.antiviral.2020.104925

[10] Si J, Gao Y, Yang Z, Liu G, Shen X, Yao Q, Zhu C. AND Logic Gate-Based Dual-Specificity DNA Circuit for Isothermal HBV rcDNA Detection. JACS Au. 2025;5(11):5778–87. 41311946 10.1021/jacsau.5c01259 PMC12648326

[11] Grimm D, Thimme R, Blum HE. HBV life cycle and novel drug targets. Hepatol Int. 2011;5(2):644–53. 21484123 10.1007/s12072-011-9261-3 PMC3090558

[12] Seok Y, Yin Q, Bai H, Bau HH. Sensitive, Single-Pot, Two-Stage Assay for Hepatitis Viruses. Anal Chem. 2022;94(3):1778–86. 35023725 10.1021/acs.analchem.1c04480 PMC11129555

[13] Vanhomwegen J, Kwasiborski A, Diop A, Boizeau L, Hoinard D, Vray M, Bercion R, Ndiaye B, Dublineau A, Michiyuki S, Manuguerra C J, Sauvage V, Candotti D, Seck A, Laperche S, Shimakawa Y. Development and clinical validation of loop-mediated isothermal amplification (LAMP) assay to diagnose high HBV DNA levels in resource-limited settings. Clin Microbiol Infect. 2021;27(12):e18589–185815. 10.1016/j.cmi.2021.03.014 33838304

[14] Liu Y, Chao Z, Ding W, Fang T, Gu X, Xue M, Wang W, Han R, Sun W. A multiplex RPA-CRISPR/Cas12a-based POCT technique and its application in human papillomavirus (HPV) typing assay. Cell Mol Biol Lett. 2024;29(1):34. 38459454 10.1186/s11658-024-00548-y PMC10921630

[15] Tian Y, Fan Z, Xu L, Cao Y, Chen S, Pan Z, Gao Y, Li H, Zheng S, Ma Y, Duan Z, Zhang X, Ren F. CRISPR/Cas13a-assisted rapid and portable HBV DNA detection for low-level viremia patients. Emerg Microbes Infect. 2023;12(1):e2177088. 36735916 10.1080/22221751.2023.2177088 PMC9946317

[16] Kachwala MJ, Smith CW, Nandu N, Yigit MV. Recombinase amplified CRISPR enhanced chain reaction (RACECAR) for viral genome detection. Nanoscale. 2022;14(37):13500–4. 36102688 10.1039/d2nr03590a PMC9623498

[17] Kachwala MJ, Smith CW, Nandu N, Yigit MV. Reprogrammable Gel Electrophoresis Detection Assay Using CRISPR-Cas12a and Hybridization Chain Reaction. Anal Chem. 2021;93(4):1934–8. 33404234 10.1021/acs.analchem.0c04949 PMC8177748

[18] Liu Z, Luo D, Ren F, Ran F, Chen W, Zhang B, Wang C, Chen H, Wei J, Chen Q. Ultrasensitive fluorescent aptasensor for CRP detection based on the RNase H assisted DNA recycling signal amplification strategy. RSC Adv. 2019;9(21):11960–7. 35517011 10.1039/c9ra01352k PMC9063470

[19] Yoon J, Lee J, Kim J, Lee SM, Kim S, Park HG. A novel ultrasensitive RNase H assay based on phosphorothioated-terminal hairpin formation and self-priming extension reaction. Biosens Bioelectron. 2024;253:116174. 38432074 10.1016/j.bios.2024.116174

[20] Nowotny M, Gaidamakov SA, Crouch RJ, Yang W. Crystal structures of RNase H bound to an RNA/DNA hybrid: substrate specificity and metal-dependent catalysis. Cell. 2005;121(7):1005–16. 15989951 10.1016/j.cell.2005.04.024

[21] Keller W, Crouch R. Degradation of DNA RNA hybrids by ribonuclease H and DNA polymerases of cellular and viral origin. Proc Natl Acad Sci U S A. 1972;69(11):3360–4. 4343966 10.1073/pnas.69.11.3360 PMC389771

[22] Fedoroff O, Salazar M, Reid BR. Structure of a DNA:RNA hybrid duplex. Why RNase H does not cleave pure RNA. J Mol Biol. 1993;233(3):509–23. 8411159 10.1006/jmbi.1993.1528

[23] Irmisch P, Ouldridge TE, Seidel R. Modeling DNA-Strand Displacement Reactions in the Presence of Base-Pair Mismatches. J Am Chem Soc. 2020;142(26):11451–63. 32496760 10.1021/jacs.0c03105

[24] Machinek RR, Ouldridge TE, Haley NE, Bath J, Turberfield AJ. Programmable energy landscapes for kinetic control of DNA strand displacement. Nat Commun. 2014;5:5324. 25382214 10.1038/ncomms6324

[25] Zhao Y, Si J, Jing S, Wang B, Xu Y, Guan J, Liu Q, Shen J, Lv M, Wang L, Zhu C. Multifunctional Framework Nucleic Acid Vehicle for Antibiotic Sensitization and Treatment of Methicillin-Resistant Staphylococcus aureus. Aggregate. 2025;6(7):e70059.

[26] Wang L, Yao Q, Guo X, Wang B, Si J, Wang X, Jing S, Yan M, Shi Y, Song G, Shen X, Guan J, Zhao Y, Zhu C. Targeted delivery of CEBPA-saRNA for the treatment of pancreatic ductal adenocarcinoma by transferrin receptor aptamer decorated tetrahedral framework nucleic acid. J Nanobiotechnol. 2024;22(1):392. 10.1186/s12951-024-02665-4 PMC11223357 38965606

[27] Song G, Dong H, Ma D, Wang H, Ren X, Qu Y, Wu H, Zhu J, Song W, Meng Y, Wang L, Liu T, Shen X, Zhao Y, Zhu C. Tetrahedral Framework Nucleic Acid Delivered RNA Therapeutics Significantly Attenuate Pancreatic Cancer Progression via Inhibition of CTR1-Dependent Copper Absorption. ACS Appl Mater Interfaces. 2021;13(39):46334–42. 34549583 10.1021/acsami.1c13091

[28] Gu J, Liang J, Tian T, Lin Y. Current Understanding and Translational Prospects of Tetrahedral Framework Nucleic Acids. JACS Au. 2025;5(2):486–520. 40017737 10.1021/jacsau.4c01170 PMC11862954

[29] Zhang T, Tian T, Lin Y. Functionalizing framework nucleic-acid-based nanostructures for biomedical application. Adv Mater. 2022;34(46):e2107820. 34787933 10.1002/adma.202107820

[30] Wang L, Guo DB, Shan YF, Sun LB, Wang R, Wang BM, Guan JY, Liu Q, Li RZ, Zhu CF, Zhao YC. Nanoengineered polymeric RNA nanoflowers for dual-pathway modulation and effective therapy for non-small cell lung cancer. *Chemical Engineering Journal* 2025, *525*.

[31] Duckett DR, Lilley DM. The three-way DNA junction is a Y-shaped molecule in which there is no helix-helix stacking. EMBO J. 1990;9(5):1659–64. 2328731 10.1002/j.1460-2075.1990.tb08286.x PMC551862

[32] Li YG, Tseng YD, Kwon SY, D’Espaux L, Bunch JS, Mceuen PL, Luo D. Controlled assembly of dendrimer-like DNA. Nat Mater. 2004;3(1):38–42. 14704783 10.1038/nmat1045

[33] Oleksy A, Blanco AG, Boer R, Uson I, Aymami J, Rodger A, Hannon MJ, Coll M. Molecular recognition of a three-way DNA junction by a metallosupramolecular helicatevol 45, pg 45, (2006). *Angew Chem Int Edit* 2006, *45* (12), 1834–1834. 10.1002/anie.200503822 16463312

[34] Sabir T, Toulmin A, Ma L, Jones AC, McGlynn P, Schroder GF, Magennis SW. Branchpoint Expansion in a Fully Complementary Three-Way DNA Junction. J Am Chem Soc. 2012;134(14):6280–5. 22329743 10.1021/ja211802z

[35] An R, Kawai H, Asanuma H, Komiyama M, Liang XG. Isothermal double-cycle catalytic system using DNAzyme and RNase H for the highly selective one-pot detection of oligonucleotides. Analyst. 2019;144(8):2773–9. 30869659 10.1039/c8an02520g

[36] Chang F, Sun YY, Yang DD, Yang WJ, Sun YY, Liu CH, Li ZP. Specific detection of RNA mutation at single-base resolution by coupling the isothermal exponential amplification reaction (EXPAR) with chimeric DNA probe-aided precise RNA disconnection at the mutation site. Chem Commun. 2019;55(48):6934–7. 10.1039/c9cc02700a 31140481

