# Supplementary material for: A toehold-triggered switchable three-way junction protective nanoprobe for RNase H-assisted HBV rcDNA detection
Source: J Nanobiotechnology. 2026 Apr 10;24:476. doi: 10.1186/s12951-026-04344-y (PMC13200495; doi:10.1186/s12951-026-04344-y)
Supplement: Supplementary file 1 — Supplementary Material 1. [file 12951_2026_4344_MOESM1_ESM.pdf]

SUPPORTING INFORMATION

A Toehold-Triggered Switchable Three-Way Junction Protective Nanoprobe for RNase H-Assisted HBV rcDNA Detection

Jingyi Si[1,3 †], Suo Lv[2†], Yifan Gao[1,3†], Zhenzhou Yang[4], Wen Liang[4], Xizhong Shen[1,3], Gang Liu[4]*, Shao Su[2]*, Changfeng Zhu[1,3]*

1 Department of Gastroenterology and Hepatology, Zhongshan Hospital, Fudan University, Shanghai, China

2 State Key Laboratory of Organic Electronics and Information Displays & Jiangsu Key Laboratory for Biosensors, Institute of Advanced Materials (IAM), Nanjing University of Posts and Telecommunications, Nanjing, China

3 Shanghai Institute of Liver Diseases, Shanghai, China

4 Key Laboratory of Bioanalysis and Metrology for State Market Regulation, Shanghai Institute of Measurement and Testing Technology, Shanghai, China

Correspondence: Gang Liu (liug@simt.com.cn), Shao Su (iamssu@njupt.edu.cn), Changfeng Zhu (zhuchangfeng@fudan.edu.cn)

† Jingyi Si, Suo Lv and Yifan Gao are contributed equally to this work.

Table S1. Sequences of the oligonucleotides used in this work.

Oligos	Sequences (5' - 3')
Probe 1 （P_1)	TGCGTGGAAGAGTATCAGTATCCGG
P_1-Q	BHQ1-TGCGTGGAAGAGTATCAGTATCCGG
Probe 2 （P_2)	CCGGATACTGATACTCCCTTTG
Template-FQ	BHQ-GAGACACAAA/rG//rG//rU//rU/CCACGCA-6-FAM
Template-F	GAGACACAAA/rG//rG//rU//rU/CCACGCA-6-FAM
Template	GAGACACAAA/rG//rG//rU//rU/CCACGCA
HBV-DNA	TGCGTGGAACCTTTGTGTCTC
SM-7	TGCGTGCAACCTTTGTGTCTC
SM-10	TGCGTGGAAGCTTTGTGTCTC
SM-14	TGCGTGGAACCTTAGTGTCTC
SM-19	TGCGTGGAACCTTTGTGTGTC

Table S2. Detailed information of all samples in this work.

Sample	Gender	Age	ALT (U/L)	AST (U/L)	HBsAg (COI)
P1	M	65	37	40	(+) 1730
P2	M	47	298	246	(+) 818
P3	M	67	54	46	(+) 1977
P4	M	55	47	35	(+) 754
P5	M	62	14	28	(+) 1909
P6	M	64	67	47	(+) 2062
P7	M	47	97	65	(+) 1296
P8	M	52	31	34	(+) 1977
P9	M	70	19	20	(+) 2040
P10	M	67	31	30	(+) 1722

Table S3. Comparison of different HBV DNA assays.

Amplification Strategy	LOD	Linear Ranges	Ref
RCA	0.5 pM	3.3-33 pM	1
exonuclease III-assisted signal amplification	10 pM	50 pM– 5 nM	2
HCR	5 pM	5 pM - 0.1 nM	3
DNA circuit	100 pM	1-20 nM	4
CRISPR-Cas12a	0.1 pM	0.1 pM-1 nM	5
DNA walking	50.4 pM	0.5nM-125nM	6
RNase-H assisted target recycling amplification	0.087 pM	0.2 pM- 5 nM	this work

Reference

1. Su, C.; Liu, Y.; Ye, T.; Xiang, X.; Ji, X.; He, Z., Rolling cycle amplification based single-color quantum dots-ruthenium complex assembling dyads for homogeneous and highly selective detection of DNA. *Anal Chim Acta* **2015,** *853*, 495-500.

2. Wu, T.; Li, X.; Fu, Y.; Ding, X.; Li, Z.; Zhu, G.; Fan, J., A highly sensitive and selective fluorescence biosensor for hepatitis C virus DNA detection based on δ-FeOOH and exonuclease III-assisted signal amplification. *Talanta* **2020,** *209*, 120550.

3. Wang, H.-B.; Zhong, Z.-T.; Zhang, T.; Zhao, Y.-D., Development of dual strip biosensors based on hybridization chain reaction and microplate strategies for signal

amplification of HBV-DNA detection. *Sensors and Actuators B: Chemical* **2020,** *310*, 127829.

4. Lv, S.; Yao, Q.; Yi, J.; Si, J.; Gao, Y.; Su, S.; Zhu, C., Leveraging Concentration Imbalance-Driven DNA Circuit as an Operational Amplifier to Enhance the Sensitivity of Hepatitis B Virus DNA Detection with Hybridization-Responsive DNA-Templated Silver Nanoclusters. *JACS Au* **2024,** *4* (6), 2323-2334.

5. Du, Y.; Ji, S.; Dong, Q.; Wang, J.; Han, D.; Gao, Z., Amplification-free detection of HBV DNA mediated by CRISPR-Cas12a using surface-enhanced Raman spectroscopy. *Anal Chim Acta* **2023,** *1245*, 340864.

6. Liang, L.; Jiang, Y. J.; Zhang, L. C.; Liu, H.; Li, Y. F.; Li, C. M.; Huang, C. Z., Rational fabrication of a DNA walking nanomachine on graphene oxide surface for fluorescent bioassay. *Biosensors and Bioelectronics* **2022,** *211*, 114349.
